# Supplementary material for: miR-4432 Targets FGFBP1 in Human Endothelial Cells
Source: Biology (Basel). 2023 Mar 16;12(3):459. doi: 10.3390/biology12030459 (PMC10045418; doi:10.3390/biology12030459)
Supplement: Supplementary file 1 [file biology-12-00459-s001.zip › Supplementary.pdf]

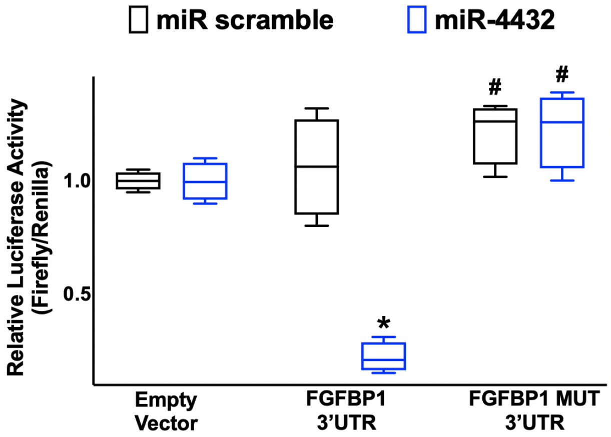

Supplementary Figure S1.

Validation of FGFBP1 targeting by miR-4432 in HUVECs.

Luciferase activity was measured 48 h after transfection, using the vector without FGFBP1 3′UTR (empty vector), the vector containing the wild-type FGFBP1 3′UTR, and the vector containing a mutated FGFBP1 3′UTR (FGFBP1 MUT); a non-targeting miRNA (miR scramble) was employed as a further control. All experiments were performed at least in triplicate; the box-and-whiskers graph indicates the medians and the 5th–95th percentiles; * $p < 0.01$ vs. miR scramble; # $p < 0.05$ vs. FGFBP1 3′UTR.
